# Among the Profession in Illinois

**Published:** 1898-06

**Authors:** 


### AMONG THE PROFESSION IN ILLINOIS.

Prof. James M. Wright, of McKillip Veterinary College, has been appointed comparative pathologist for the Academy of Science of Chicago.

Dr. E. L. Quitman, of Chicago, considers a carefully adjusted mouth one of the fine arts of veterinary surgery.

Dr. F. S. Schoenleber, of Chicago, says that a graduate not well-drilled or equipped in anatomy will never make a good surgeon or a successful practitioner.

Dr. F. T. McMahon, of the McKillip Veterinary College, fully realizes the importance of a thorough course in meat- and milk-inspection in these advancing times.

Dr. A. H. Baker, of Chicago, scans daily the rise and fall of the stock-market, and takes keen interest in the present advance in price and demand for black diamonds.

Dr. L. A. Merillat, of McKillip Veterinary College, has secured the position of veterinary inspector for the purchase of army horses at the Chicago market.

Dr. C. H. Zink, a Philadelphian, was so enthusiastic over the charms of the "Windy City" that the ink was scarcely dry upon his sheepskin, obtained at McGill University, Montreal, before he hied himself to the city of his adoption.

Dr. James Robertson, of Chicago, associates with his work that of horseshoeing, and takes a keen interest in all advances in the shoeing art.

Dr. James M. Wright, of Chicago, finds the attractions of canine practice the chief end of his veterinary studies and professional employment.

Dr. M. R. Trumbower, of Sterling, after years of labor in the field of comparative medicine, has finally drifted into the specialty of human medicine, where he hopes for larger fees and greater renown.

Dr. L. A. Merillat, of Chicago, gives much time and attention to veterinary dentistry, and his contributions to the study of this subject have proven of much interest.

Dr. C. A. White, of Chicago, fills the rôle of assistant to Dr. Joseph Hughes, of the Chicago Veterinary College.

Dr. John B. Boomer, graduate of the McKillip Veterinary College, class of 1897, is one of the practising staff of the hospital of that institution.

Dr. S. S. Baker, of Chicago, finds time from the exacting duties of routine practice to indulge in association privileges, and is a regular at State Association conventions.

Dr. A. E. Flowers, Professor of Dental Surgery at the Chicago Veterinary College, fills the rôle of professor of comparative anatomy in the Northwestern University.

Dr. Charles L. Widmeyer, graduate of Ontario Veterinary College, class of 1894, holds a position on the practical staff of instructors of McKillip Veterinary College.

Dr. A. S. Nesbitt, a graduate of the class of 1894, of the Chicago Veterinary College, holds the position of assistant to Prof. A. H. Baker, of that institution.

Dr. Joseph Hughes, of Chicago, finds no part of his work more enjoyable than the selection of a saddle-horse.

Dr. A. S. Alexander, of Evanston, finds recreation and enjoyment in answering veterinary queries through the columns of the *Breeders' Gazette.*

Dr. W. S. Devoe, of Chicago, finds but one attraction in the veterinary world, and that is the search for contagious and infectious diseases.

Dr. W. J. Martin, of Kankakee, finds his keenest delight in searching out the mysteries of some intricate and unusual case.

Dr. B. A. Pierce, of Chicago, finds the running down of glandered horses a serious task at times.

Dr. Albert Babb, of Springfield, finds association work congenial, and has been a strong factor in the growth of State association.

Dr. John Casewell, of Chicago, yearns for the return of State work, which for many years formed the most enjoyable part of his occupation.

Dr. G. W. Turner, of Highwood, finds army duties congenial, but yearns for the time when the veterinarian will be accorded rank and be made a commissioned officer.

Dr. R. J. Withers, formerly of Chicago, has never found the shores of California as congenial a resting-place as the "Windy City."

Dr. Louis P. Cook, of East St. Louis, has not satisfied himself yet as to whether the soil of Illinois will be as congenial as that of the Empire State, from which he recently moved.

Dr. Harri H. Dell, of Sullivan, was a strong association member at College, and it is to be hoped will be equally zealous in the "Prairie State," where he located after graduation.

Dr. B. A. Pierce, Assistant State Veterinarian, represents the State Board of Agriculture for inspection of all horses consigned for sale.

Dr. Lawrence Campbell, of Chicago, could not long remain out of the harness of secretary, which is so congenial to his tastes, and in which he has displayed marked ability.

Dr. C. E. Hollingsworth, of La Salle, is an active association member and a frequent contributor to the programme.

Farmer Miles, of Princeton, makes fewer pilgrimages over the country than he did some years ago.

Dr. Eugene Sullivan, of Chicago, considers the official work as city veterinarian a special opportunity for demonstrating the great need of veterinary services for the care of the city livestock.

Dr. D. J. McIntosh, of Urbana, finds the study of the diseases of the porcine tribe specially interesting.

Dr. M. H. McKillip, of Chicago, finds special methods of surgery attractive and specially applicable in veterinary practice.

Dr. H. W. Hawley, of Chicago, thinks that the vision of many veterinary surgeons is too acute when passing upon the imperfections and soundness of the horse.

Dr. Hugh Thompson, formerly of Manhattan, has now located at Shabbona.

